# Implications for selecting local excision in locally advanced rectal cancer after preoperative chemoradiation

**DOI:** 10.18632/oncotarget.3418

**Published:** 2015-03-23

**Authors:** Juefeng Wan, Kaitai Liu, Ji Zhu, Guichao Li, Zhen Zhang

**Affiliations:** ^1^ Department of Radiation Oncology, Fudan University Shanghai Cancer Center, Shanghai, China; ^2^ Department of Oncology, Shanghai Medical College, Fudan University, Shanghai, China; ^3^ Department of Radiation Oncology, Lihuili Hospital, Ningbo Medical Center, Ningbo, China

**Keywords:** rectal cancer, local excision, positive lymph nodes, chemoradiotherapy, seer

## Abstract

Local excision may offer the possibility of organ preservation for the management of locally advanced rectal cancer after neoadjuvant chemoradiotherapy (CRT). However, the oncological outcomes of this strategy have been largely associated with the risk of nodal metastases. In this study, Surveillance, Epidemiology, and End Results Program (SEER)-registered rectal cancer patients, and patients from Fudan University Shanghai Cancer Center (FUSCC) after preoperative chemoradiation were combined to analyze the incidence of lymph node metastasis. The results showed that there was a high risk for residual lymph node metastasis among patients even with complete pathologic response of primary tumor after preoperative CRT (12.6–13.2%). However, in the selected group of patients with pre-CRT MRI staging cN0 rectal cancer, there was only one ypN+ case (3.3%) in ypT0–1 group. These results suggest that pre-CRT MRI staging cN0 patients achieved ypT0–1 of bowel wall tumor may be suitable for local resection.

## INTRODUCTION

Neoadjuvant chemoradiotherapy (CRT) followed by total mesorectal excision (TME) is a standard treatment in patients with locally advanced rectal cancer (LARC) [1–5]. However, radical surgery is associated with significant morbidity, especially in cases of low rectal cancer [6, 7]. Local excision may offer the possibility of organ preservation for the management of select patients after neoadjuvant chemoradiation.

However, the oncological outcomes of this strategy have been largely associated with the risk of nodal metastases. Therefore, cautious and strict patient selection is crucial in this approach. Ideal candidate tumors for this treatment approach should be restricted to the bowel wall and harbor minimal risk for lymph nodes (LNs) metastases. Given the growing importance of lymph node metastases in the management of local excision, we designed our study to specifically assess the incidence of the positive lymph nodes in patients with locally advanced rectal cancer after chemoradiation by analyzing the Surveillance, Epidemiology, and End Results (SEER)-registered database. Moreover, because SEER data lacks information on pre-CRT clinical stage, neoadjuvant chemoradiotherapy (NCRT) methods, we further clarified these relevant issues in another set of patients with locally advanced rectal cancer from the Fudan University Shanghai Cancer Center (FUSCC).

## RESULTS

### SEER database patient characteristics

A total of 12,682 eligible patients during the 8-year study period were indentified, including 7,982 male and 4,700 female patients. There were 114 patients (0.9%) with ypT0 stage, 1091 patients (8.6%) with ypT1 stage, 1989 patients (15.7%) with ypT2 stage, and 9488 patients (74.8%) with ypT3–4 stage rectal cancer. Patient demographics and pathological features are summarized in Table 1. The proportion of well differentiation (Grade I) gradually decreased from ypT0 to ypT3/4 (10.5% to 6.1%).

**Table 1:** Patient characteristics from SEER database

	ypT0		ypT1		ypT2		ypT3–4	
Variable	n	%	n	%	n	%	n	%
**Sex**								
Male	74	64.9	692	63.4	1297	65.2	5919	62.4
Female	40	35.1	399	36.6	692	34.8	3569	37.6
**Age**								
<50	20	17.5	195	17.9	377	19	1933	20.4
≥50	94	82.5	896	82.1	1612	81	7555	79.6
**Race**								
White	99	86.8	910	83.4	1604	80.6	7764	81.8
Black	8	7	97	8.9	188	9.5	729	7.7
Other	7	6.2	84	7.7	197	9.9	995	10.5
**Pathological grading**								
Grade I	12	10.5	89	8.2	127	6.4	581	6.1
Grade II	63	55.3	707	64.8	1459	73.4	6474	68.2
Grade III	11	9.6	95	8.7	190	9.6	1273	13.4
Grade IV	2	1.8	5	0.5	8	0.4	111	1.2
Unknown	26	22.8	195	17.8	205	10.2	1049	11.1
**Histotype**								
Adenocarcinoma	109	95.6	1058	97	1911	96.1	8685	91.5
Mucinous/Signet ring cell	5	44	33	3	78	3.9	803	8.5
**LNs examined**								
Median	6		8		11		12	
Rang	1–21		1–25		1–28		1–35	

Abbreviations: LNs, lymph nodes.

### Incidence of positive lymph nodes

Overall, 5649(44.5%) patients had lymph node metastasis. Patients with higher ypT categories following chemoradiotherapy were more likely to also have positive ypN status (*P* < 0.001). By ypT stage, the numbers of ypN+ tumors were 15 (13.2%) for ypT0, 186 (17 %) for ypT1, 618 (31%) for ypT2, and 4830 (50.9%) for ypT3/4. Patients were categorized into two groups based on the identification of lymph nodes metastasis: ypN0 and ypN+ (Table 2).

**Table 2:** Association of positive nodes with clinical/pathologic variables from SEER database

	LN–		LN+		
Variable	n	%	n	%	*p*
**Sex**					
Male	4452	55.8	3530	44.2	0.706
Female	2581	54.9	2119	45.1	
**Age**					
<50	1182	46.8	1343	53.2	<0.001
≥50	5851	57.6	4306	42.4	
**Race**					
White	5804	55.9	4573	44.1	0.305
Black	569	55.7	453	44.3	
other	660	51.4	623	48.6	
**Pathological grading**					
Grade I	449	61.7	310	38.3	0.017
Grade II	4943	56.8	3760	43.2	
Grade III	680	43.3	889	56.7	
Grade IV	42	33.3	84	66.7	
unknown	869	58.9	606	41.1	
**Histotype**					
Adenocarcinoma	6663	56.6	5100	43.4	<0.001
Mucinous/Signet ring cell	370	40.3	549	59.7	
**ypT**					
0	99	86.8	15	13.2	<0.001
1	905	83	186	17	
2	1371	69	618	31	
3/4	4658	49.1	4830	50.9	
**LNs examined**					
Median	10		13		<0.001
Rang	1–27		1–35		

Abbreviations: LNs, lymph nodes.

### Study of potential associations

To discard potential bias in the detection of pathologically positive LNs, we studied possible associations between patient and tumor characteristics (Table 2). Sex did not correlate with ypN+ (*P* = 0.706), because 3530 (44.2%) of 7982 male patients had ypN+ compared with 2119 (45.1%) of 4700 female patients. As seen in Table 2, the rate of ypN+ did differ significantly between adenocarcinoma (43.4%) and mucinous/signet ring cell (59.7%; *P* < 0.001). In addition, the race was not found to be significantly associated with the incidence of ypN+ (*P* = 0.305).

### Evaluating the seer database outcomes using the fuscc set

The above results should be treated with caution as they might be biased by confounding factors, such as pre-CRT stage and concurrent chemotherapy. To evaluate the reliability of SEER results, we studied relevant issues in 517 eligible patients from the FUSCC. Patient demographics and pathological features are summarized in Table 3.

**Table 3:** Demographic and clinical features of patients with rectal cancer from Fudan University Shanghai Cancer Center

	ypT0		ypT1		ypT2		ypT3–4	
Variable	n	%	n	%	n	%	n	%
**Sex**								
Male	78	70.3	23	74.2	74	62.2	185	72.3
Female	33	29.7	8	25.8	45	37.8	71	27.7
**Age**								
<50	40	36	8	25.8	37	31.1	77	30.1
≥50	71	64	23	74.2	82	68.9	179	69.9
**Baseline stage**								
II	23	20.7	7	22.6	19	16	43	16.8
III	88	79.3	24	77.4	100	84	213	83.2
**Distance from anal verge**								
≤5cm	73	65.8	20	64.5	76	63.9	142	55.5
>5cm	38	34.2	11	35.5	43	36.1	114	44.5
**LNs examined**								
Median	9		9		10		10	
Range	1–24		4–20		2–27		1–28	
**CCT**								
fluorouracil alone	39	35.1	11	35.5	36	30.3	112	43.8
FBCR	72	64.9	20	64.5	83	69.7	144	56.2

Abbreviations: LNs, lymph nodes; FBCR, fluorouracil-based combination regimens; CCT, concurrent chemotherapy.

### Incidence of positive LNs

In 193 of 517 patients (37.3%), routine pathologic analysis of the resected specimen revealed positive LN involvement. Patients with higher ypT categories following chemoradiotherapy were more likely to also have positive ypN status (*P* < 0.001). By ypT stage, the numbers of ypN+ tumors were 14 (12.6%) for ypT0, 6 (19.4 %) for ypT1, 38 (31.9%) for ypT2, and 135 (52.7%) for ypT3/4. In addition, we assessed the rate of positive LN involvement according to the pre-CRT MRI staging. Our findings showed that the proportion of lymph node metastasis in ypT0–1 cases was 17% among pre-CRT MRI staging cN+ patients. In the selected group of patients with pre-CRT MRI staging cN0 rectal cancer, there was only one ypN+ case (3.3%) which was tumor nodules rather than lymph node in ypT0–1 group. Patients were categorized into two groups based on the identification of lymph node metastasis: ypN0 and ypN+ (Table 3).

### Study of potential associations

To discard potential bias in the detection of pathologically positive LNs, we studied possible associations between patient and tumor characteristics, concurrent chemotherapy regimens (Table 4). Distance from the anal verge did not correlate with ypN+ (*P* = 0.691), because 114 (36.7%) of 311 patients with tumors located 0 to 5 cm from the anal verge had ypN+ compared with 79 (38.3%) of 206 patients with tumors located 6 to 12 cm from the anal verge. As seen in Table 4, the rate of ypN+ did not differ significantly between different concurrent chemotherapy (fluorouracil alone or fluorouracil-based combination regimens) (*P* = 0.697).

**Table 4:** Association of positive nodes with clinical/pathologic variables from fudan university shanghai cancer center

	LN–		LN+		
Variable	n	%	n	%	P
**Age (yr)**					
Median	56		56		0.002
Range	26–77		20–82		
**Gender**					
Male	236	65.6	124	34.3	0.04
Female	88	56.1	69	43.9	
**Distance from anal verge**					
Median	5		5		0.691
Range	1–12		0–12		
≤5 cm	197	63.3	114	36.7	
>5 cm	127	61.7	79	38.3	
**CCT**					
fluorouracil alone	122	61.6	76	38.4	0.697
FBCR	202	63.3	117	36.8	
**YPT**					
0	97	87.4	14	12.6	<0.001
1	25	80.6	6	19.4	
2	81	68.1	38	31.9	
3/4	121	47.3	135	52.7	
**ypT, pre-CRT stage (N0)**					
0	22	95.7	1	4.3	0.017
1	7	100	0	0	
2	17	81	4	19	
3/4	30	71.4	12	28.6	
**ypT, pre-CRT stage (N+)**					
0	75	85.2	13	14.8	<0.001
1	18	75	6	25	
2	64	65.3	34	34.7	
3/4	91	42.5	123	57.5	

Abbreviations: CRT, chemoradiotherapy; LN, lymph node; FBCR, fluorouracil-based combination regimens; CCT, concurrent chemotherapy.

## DISCUSSION

Chemoradiotherapy (CRT) followed by total mesorectal excision was considered the standard of care in the treatment of locally advanced rectal cancer since it was proven to be beneficial in reducing the rate of local recurrence and toxicity [1–5]. In order to avoid the potential morbidity and impaired long-term functional outcomes associated with radical resection, there has been an increasing interest for organ-preserving strategies with local excision in the management of patients with rectal cancer and good response to neoadjuvant CRT.

Local excision of rectal tumors is a technique with significant lower morbidity and mortality rates, compared with standard radical surgery [8, 9]. Stipa et al. evaluated the long-term clinical outcome of a selected group of 43 patients who underwent local excision with transanal endoscopic microsurgery after chemoradiation. In the ypT0 group, no local and distal recurrences were observed. In the ypT1–3 group, local recurrence was 10/30 (33%) [10]. In addition, Noh and colleagues reported the outcome of local excision following preoperative chemoradiotherapy for cT2 rectal cancer. The 5-year disease-free survival was higher in patients with ypT0 (90%) than in patients with ypT1–2 (69%, *p* = 0.1643) [11]. Moreover, Belluco et al. conducted a study on 29 patients treated by local excision, comparing patients with ypCR to patients with no ypCR, 5-year local recurrence-free survival was 92.9% vs. 66.7% (*P* = 0.047) [12]. Therefore, these studies suggested that TEM may have a curative role in the case of complete response to CRT.

Despite these several studies had reported that local excision in patients who showed a good response to CRT had acceptably low rates of local recurrence and long-term survival outcomes compared with radical surgery. However, the issue of local resection following preoperative CRT has been addressed by few studies, which are limited by the low numbers and short follow-up [10–13].

Although response at the primary tumor site within the bowel may provide insight into the status of residual disease within the mesorectum, one of the uncertain facts which could not be ignored when conducting local excision is the status of the mesorectal lymph nodes. Some studies have confirmed that there can be differential responses between the primary tumor and the mesorectal lymph nodes [14, 15]. When nodal involvement is understaged and patients undergo local excision, the prognosis is poorer. Park and colleagues determined the rate of residual lymph node involvement following neoadjuvant chemoradiotherapy among patients with ypT0–2 residual bowel wall tumor [16]. Among all 406 ypT0–2 patients, 66 (16.3%) had lymph node metastasis: 20.8% among ypT2, 17.1% among ypT1, and 9.1 % among ypT0 patients. Local recurrences occurred more frequently in ypN+ vs ypN0 patients (2.0% vs. 5.5%; *p* = 0.038). Recurrence-free survival was 87.5% among ypT0–2N0 and 83.6% forypT0–2N+ (*P* = 0.28). With the T staging, lymph node metastasis rate also increased. In the present study, the SEER data showed that lymph node metastasis was 31% among ypT2, 17% among ypT1, and 13.2 % among ypT0 patients. Patient data from FUSCC showed that the incidence of lymph node involvement was 12.6% in patients developing mural pCR (ypT0) compared to 19.4% for ypT1 and increased further to 31.9% for ypT2 tumors which was comparable to the SEER data. These data showed that the incidence of lymph node involvement was more than 10% in patients with complete response of primary tumor.

Recently, a randomized trial of patients with cT2N0 following preoperative CRT to either TME or local excision (using transanal endoscopic microsurgery, TEM) suggested equivalent local disease control with both techniques [17]. The risk of lymph node metastases after CRT is already minimized when proper staging at baseline shows cN0. Based on this, perhaps baseline lymph node staging may play a significant role in predicting the risk of lymph node metastases after CRT. Guillem et al. showed the incidence of positive LNs in patients with pre-CRT stage cT3N0 after CRT: ypT0, 3%; ypT1, 7%; ypT2, 20%; ypT3–4, 36% (*P* = .001) which was significantly lower than patient with cT3, 4N+ [18]. In the present study, we assessed the rate of positive LN involvement according to the pre-CRT MRI staging. Our findings showed that the proportion of lymph node metastasis in ypT0–1 cases was 17% among pre-CRT MRI staging cN+ patients. In the selected group of patients with pre-CRT MRI staging cN0 rectal cancer, there was only one ypN+ case (3.3%) which was tumor nodule rather than lymph node in ypT0–1 group.

MRI has been used to delineate locally stage non-irradiated rectal cancers. Recently, several studies have shown that the use of MRI improves the overall T staging accuracy for rectal cancer with accuracy rates of 86%–95% [19–21]. Regarding LN assessments with MRI, the sensitivity and specificity are 75%–89% and 71%–98%, respectively [21–25]. Restaging MRI is performed after CRT to restage rectal cancer to identify the response of chemoradiation. Lee et al. conducted a study to evaluate the efficacy of restaging MRI for predicting the pathologic stage in rectal cancer after CRT. Pathologic T classification matched the post-CRT MRI findings in 97 (64.7%) of 150 patients and pathologic N classification matched the post-CRI MRI findings in 85 (56.6%) of 150 patients [26]. In addition, Park et al. determined whether preoperative MRI could detect lymph node metastases accurately in the node-by-node analysis [27]. Of the 341 nodes harvested, 120 were too small (<3 mm) to be depicted on magnetic resonance images, and 18 of these contained metastasis (15%). Preoperative MRI revealed anode-by-node sensitivity and positive predictive value of 58.0%, and 61.7%. Therefore, preoperative MRI has low accuracy for the prediction of the pathologic T and N classifications in rectal cancer patients who received preoperative CRT.

Our study has several limitations that deserve mention. First, although the present study is a large population-based study, the SEER database does not include information regarding the administration of CRT and the quality of surgical care or pathological technique, and all of these factors may affect positive LNs harvest. Second, it is a retrospective analysis and was therefore limited by the bias inherent in this type of analysis. However, given that the study patients were consecutive, offering a non-selected series of T3,4 and/or N+ rectal cancers, we believe that our results do not reflect a bias toward patients.

In conclusion, given use of ypT stage only to stratify patients for local excision is partly unsafe, organ-preserving strategies for these patients will need to consider baseline MRI imaging in addition to CRT response to identify eligible patients. Our study demonstrates that there was a high risk for residual lymph node metastasis among patients even with complete pathologic response of primary tumor after preoperative CRT (12.6–13.2%). But if cT3,4N0 patients whom be classified according to baseline MRI imaging achieved ypT0–1 of bowel wall tumor, the rate of positive LN involvement was clinically acceptable (3.3%, tumor nodule, actually). This group of patients may be suitable for local resection. Considering that this study is a retrospective analysis, we need further prospective studies to verify.

## MATERIALS AND METHODS

### Patient selection in the SEER database

The SEER, a population-based reporting system, was surveyed for the retrospective collection of data used in the analysis. The SEER program collects and publishes cancer incidence and survival data from 18 population-based cancer registries, covering >25% of the US population. Because no personal identifying information was used in the analysis, this study was granted an exemption from the Institutional Review Board of the study institution on March 30, 2012.

Cases of rectal cancer (C20.9 Rectum, NOS) from 2004 to 2011 were extracted from the SEER database (SEER*Stat 8.1.5) according to the Site Recode classifications with limitation to radiation prior to surgery and radiation preoperatively and post-surgery. Histological type were limited to adenocarcinoma (ICD-03, 8140/3, 8210/3, 8261/3, 8263/3), mucinous adenocarcinoma (ICD-03, 8480/3), and signet ring cell carcinoma (ICD-03, 8490/3). We selected this range because American Joint Committee on Cancer (AJCC) TMN stage was available since 2004. Other exclusion criteria were as follows: no LNs examined pathologically, synchronous distance metastases, and patients with unknown TNM stage.

### Patient selection in the FUSCC

The Fudan University Shanghai Cancer Center Ethics Review Board approved the study. Preoperative chemoradiation was performed as standard treatment of LARC since 2006, so we performed a retrospective consecutive cohort study of locally advanced rectal cancer patients with preoperative chemoradiation at FUSCC between 2006 and 2013. Patients were identified from our institutional patient colorectal cancer database. Patients with no LNs examined pathologically, synchronous distance metastases, and unknown TNM stage were excluded.

### Treatment details

Pretreatment clinical stage was assessed on the basis of MRI. All pretreatment biopsies were reviewed and diagnoses confirmed by Shanghai Cancer Center gastrointestinal pathologists. All patients also underwent full colonoscopic evaluation to exclude synchronous tumors, as well as digital rectal examination and proctoscopy to identify the tumor distance from the anal verge. Patients were treated with chemoradiotherapy with a median radiotherapy dose of 50 Gy and concurrent fluorouracil-based chemotherapy. Surgery generally was performed 6 to 8 weeks following completion of chemoradiotherapy and included low anterior resection, or abdominoperineal resection using total mesorectal excision (TME) principles. Adjuvant chemotherapy consisted of FOLFOX, XELOX, or Capecitabine for a period of 4 to 5 months was recommended for all medically fit patients following resection. Standard pathologic tumor staging of the resected specimen was performed after resection in accordance with the guidelines of the College of American Pathologists, with histopathologic diagnosis performed by dedicated gastrointestinal cancer pathologists. The gross tumor volume was entirely embedded and serially sectioned for hematoxylin and eosin staining and microscopic evaluation. Postoperative follow-up consisted of routine physical examination with carcinoembryonic antigen measurement and cross-sectional imaging every 3–6 months for the first 2 years after completion of treatment and every 6–12 months for 2 additional years thereafter. CT scans of the chest, abdomen and pelvis, full colonoscopic evaluation, and/or positron emission tomography (PET) were immediately performed if any symptom of disease occurred or elevated tumor marker levels were detected.

### Statistical analysis

Associations between LN positivity and clinical/pathologic variables were examined using Fisher's exact test for categoric variables, an exact version of the Mantel-Haenszel test for trend for ordinal variables, and the Wilcoxon test for continuous variables. The statistical test was two sided and *P* < 0.05 was considered statistically significant. PASW Statistics 13 (SPSS Inc., Chicago, USA) was used for the statistical analysis.
